# Evaluation of the Soft Tissue Changes after Rapid Maxillary Expansion Using a Handheld Three-Dimensional Scanner: A Prospective Study

**DOI:** 10.3390/ijerph18073379

**Published:** 2021-03-24

**Authors:** Ali Alkhayer, Roland Becsei, László Hegedűs, László Párkányi, József Piffkó, Gábor Braunitzer, Emil Segatto

**Affiliations:** 1 Craniofacial Unit, Department of Oral & Maxillofacial Surgery, University of Szeged, Tisza Lajos krt. 97, 6722 Szeged, Hungary; ali.alkhayer@hotmail.com (A.A.); becsei.roli@gmail.com (R.B.); hlac929@gmail.com (L.H.); 2 Department of Periodontology, Faculty of Dentistry, University of Szeged, Tisza Lajos krt. 64-66, 6720 Szeged, Hungary; parkanyilaci@gmail.com; 3 Department of Oral & Maxillofacial Surgery, Faculty of Medicine, University of Szeged, Kálvária Sugárút 57, 6725 Szeged, Hungary; piffkojozsef@gmail.com; 4 DicomLAB Dental, Ltd., Szent-Györgyi Albert u. 2, 6726 Szeged, Hungary; braunitzergabor@gmail.com

**Keywords:** rapid maxillary expansion, malocclusion, facial soft tissue, three-dimensional imaging

## Abstract

Facial soft tissue esthetics is a priority in orthodontic treatment, and emerging of the digital technologies can offer new methods to help the orthodontist toward an esthetic outcome. This prospective study aimed to assess the soft tissue changes of the face after six months of retention following Rapid Maxillary Expansion (RME). The sample consisted of 25 patients (13 females, 12 males, mean age: 11.6 years) who presented with unilateral or bilateral posterior crossbite requiring RME, which was performed with a Hyrax expander. 3D facial images were obtained before treatment (T_0_) and at the end of a six-month retention period after the treatment (T_1_) using a structured-light 3D handheld scanner. Linear and angular measurements were performed and 3D deviation analyses were done for six morphological regions of the face. Significant changes in various areas of the nasal and the upper lip regions were observed. Based on the results of the study and within the limitations of the study, RME with a Hyrax expander results in significant morphological changes of the face after a six-month retention period.

## 1. Introduction

Rapid maxillary expansion (RME) is routinely used to eliminate skeletal maxillary transversal deficiency, particularly in patients with posterior crossbite, moderate crowding, and sleep apnea disorders [1,2,3,4], to achieve apical opening of the maxillary base along the mid-palatal suture.

The approach basically relies on the forces generated by means of the RME appliances, which can mechanically separate the maxillary segments at the mid-palatal suture [3,4,5]. By this treatment, the following can be achieved: correction of the skeletal transversal deficiency and gain of space in the dental arch [6], improved smile by reducing the buccal corridors [7] and expansion of the airway [8].

RME has an influence on the morphology of the soft tissues of the face as well. It is in the patients’ best interest that such changes are understood [9]. Still, the area is surprisingly under-researched and several questions are unclarified. 

Until recently, changes in the soft tissue envelope and the underlying skeletal structures following RME have been examined mainly through two-dimensional (2D) imaging techniques, mostly lateral cephalometric images, anteroposterior graphs, and photogrammetric analyses [10,11,12]. However, these methods suffer from superimposition and magnification problems, which can be avoided using three-dimensional (3D) imaging.

Of the three-dimensional imaging methods, cone-beam computed tomography (CBCT) is becoming increasingly popular. However, soft tissues are poorly represented using this technique [13].

Therefore, noncontact optical scanning devices were introduced as 3D imaging techniques for soft tissue visualization like laser surface scanning and stereophotogrammetry [14]. These non-invasive approaches are thought to allow images to be captured at short intervals without exposing the patient to the radiation, which makes them an appealing option [14]. However, these devices are often bulky and also rather expensive, which are clearly deterring factors [15].

By using a handheld 3D structured-light scanners, texture and color information of the face can be promptly obtained in high resolution without radiation. Further advantages include short scan time, portability, ease of operation, and reasonable cost [16]. Jung et al. found that the accuracy of structured light systems compares to that of direct anthropometric measurements and concluded that it is a reliable approach for a facial soft tissue assessment [17].

Most of the previous studies that utilized 3D facial scanners to evaluate soft tissue changes following RME have examined deviations at specific points and calculated linear and angular measurements [18,19]. However, it is more accurate to predict changes within the whole complex structures on a 3D basis rather than only between specified points. Having recognized that, the aim of our prospective study was to adopt a comprehensive examination of soft tissue in various morphological regions of the face after six months of retention following RME, based on structured-light scanning. 

## 2. Materials and Methods

This prospective study was approved by the Human Investigation Review Board, University of Szeged, Albert Szent-Györgyi Clinical Centre (No. 151/2019-SZTE).

### 2.1. Study Sample

Patients in need of upper arch expansion were recruited from among the patients of the Craniofacial Unit, Department of Oral and Maxillofacial Surgery, Albert Szent-Györgyi Clinical Center, Szeged, Hungary, between January 2019 and January 2020. The inclusion criteria included maxillary transverse deficiency, assessed both clinically and radiographically, which is associated with either unilateral or bilateral posterior crossbite and/or dental crowding. Exclusion criteria included the history of trauma or previous orthodontic treatment, and patients with physical and psychological limitations and/or craniofacial anomalies.

The sample size was calculated based on the findings of Kim et al. [20], and the analysis was performed with G*Power software (Franz Faul, Universität Kiel, Germany) Version 3.1.9.4, based on the assumption of the Wilcoxon rank test. A sample size of 23 patients was predicted to provide 80% of power with a 5% error of probability. A total of 25 patients (13 females and 12 males) with a mean age of 11.6 years (range: 8.1–14.4 years) were enrolled in our study.

The 3D facial images were acquired immediately before the appliance was cemented (T_0_) and at the end of the 6-month retention phase (T_1_), using a structured-light 3D handheld scanner (Artec Eva^TM^; Artec Group, Luxembourg). A 6-month retention period was chosen to control for growth, which could have interfered with the results in case of a longer period. 

The proposed scanner uses structured light scanning technology to accurately capture in a point-and-shoot manner up to 16 frames per second and each frame is a 3D image. These frames are aligned automatically in real-time while providing high resolution (up to 0.5 mm) and high accuracy (up to 0.1 mm). All images were taken with the head in a natural head position and with a relaxed lip posture [21]. To reach the natural head balance, subjects were seated in a back-supported and vertically adjustable chair. They were asked to turn their heads forward and backward with decreasing amplitude until they reached a relaxed position [22]. Then they were told to look straight ahead to a point on the wall in front of them at eye level.

### 2.2. Clinical Protocol

Following upper and lower alginate impressions, a Hyrax-type expander was constructed with 4 bands, palatal stainless-steel bars of 1.0-mm diameter, and a jackscrew (Forestadent, Pforzheim, Germany) with stainless steel extensions soldered to the palatal surfaces of each pair of bands. The activation of the jackscrew was for each quarter turn equivalent to 0.25 mm.

**Figure 1:**
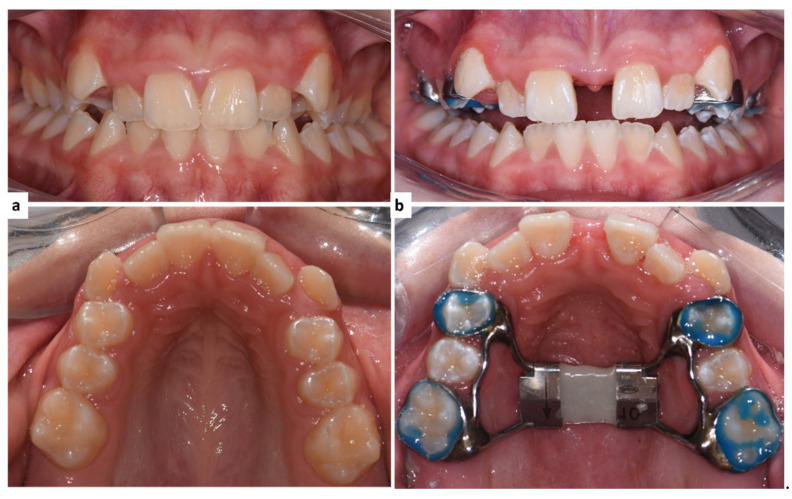
Intraoral photographs of a patient. (**a**) Before the cementation of the Hyrax expander. (**b**) After the expander was blocked for retention.

The parents were instructed to activate the screw 2 turns per day (0.5 mm) and the patients were recalled on a weekly basis of the expansion period (2 to 3 weeks). The expansion was stopped when the palatal cusp of the upper molars was touching the buccal cusp of the lower molars [23]. Then the appliance was kept in for the retention period (6 months), and the jackscrews were blocked with a composite to prevent relapse (Figure 1).

The expansion of the Hyrax jackscrew was measured for each patient. Dental cast models were also made prior to expander cementation and after the retention period. The distance between the mesial buccal cusp of the right and left upper first molar was measured before and after the expansion and the difference was calculated.

After removal of the Hyrax appliance, we continued the treatment for all our patients using a fixed appliance (self-ligating multibracket appliance, Roth prescription).

### 2.3. Data Processing and Measurements

**Figure 2:**
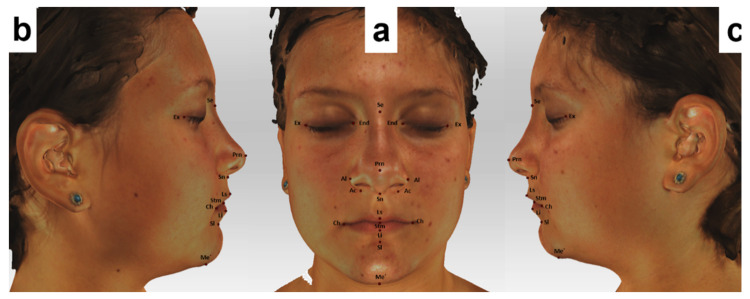
Landmarks used in our study located on the 3D-facial images. (**a**) Frontal view (**b**) and (**c**) lateral views.

**Figure 3:**
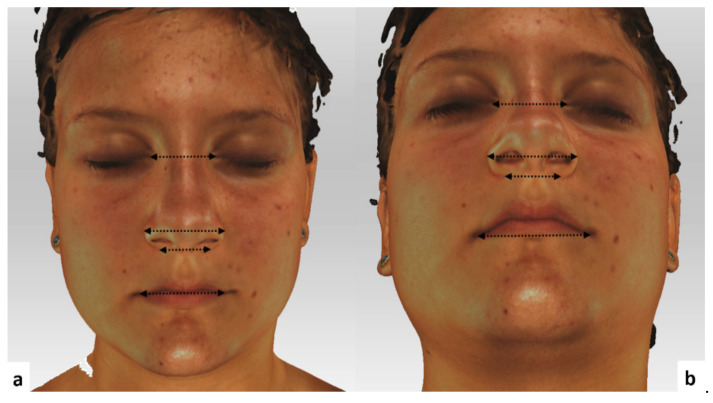
Linear measurements used in our study. (**a**) Frontal view. (**b**) Base view.

**Figure 4:**
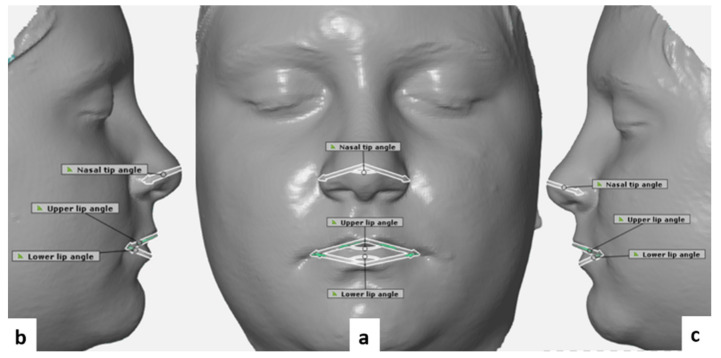
Angular measurements used in our study. (**a**) Frontal view (**b**) and (**c**) lateral views.

In total, 18 landmarks (5 bilateral and 8 unilateral: Table 1, Figure 2) were defined according to the literature [24,25]. In addition, 4 linear and 3 angular measurements were performed directly on the 3D facial images using Artec Eva V.12 (Figure 3 and Figure 4).

**Figure 5:**
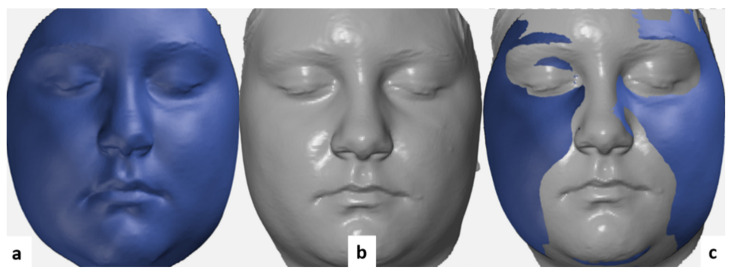
The best-fit method used in our study. (**a**) T_0_ mesh. (**b**) T_1_ mesh. (**c**) The final mesh aligned.

For the 3D deviation analysis, the 3D facial images were transferred into a reverse engineering software (GOM Inspect Evaluation Software, Capture 3D, Inc, Santa Ana, CA, USA) and polygon meshes were created in a stereolithography (STL) format. The hair, ears, and the below-neck region were removed. The images obtained at the T_0_ time point were aligned with the images taken at T_1_ using the best-fit method, as described by Dindaroglu et al. [26] (Figure 5). 

**Figure 6:**
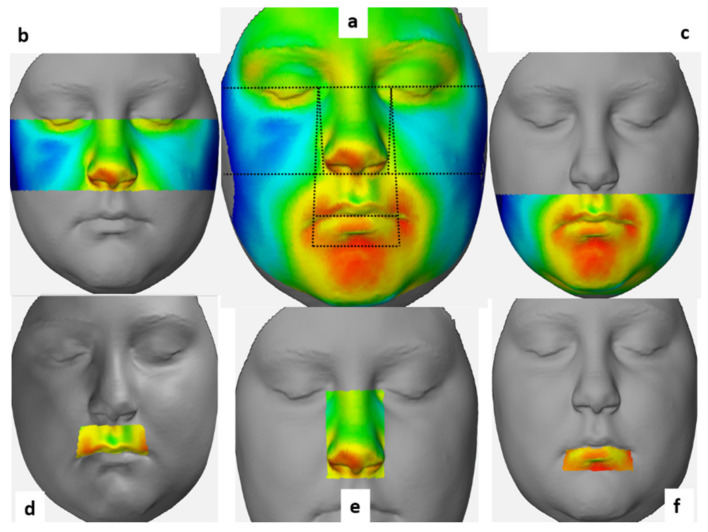
The morphological regions and their reference lines used in our study. (**a**) Total face region with the reference lines used. (**b**) Upper face region. **(c)** Lower face region. (**d**) Upper lip region. (**e**) Nose region. (**f**) Lower lip region.

Negative values indicate that T_1_ images were located behind the T_0_ images (blue shades), whereas positive values indicate that T_1_ images were located in front of the T_0_ images (red shades). To create morphological regions, eight lines passing through different points specified on the face were determined and a 3D deviation analysis was made in six morphological regions [26] (Table 2**,** Figure 6).

**Figure 7:**
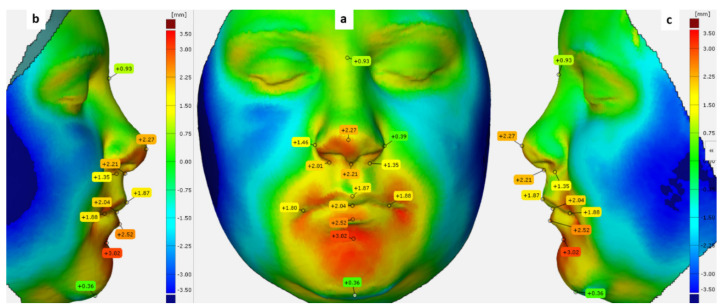
Deviation labels of the facial soft tissue landmarks used in our study. (**a**) Frontal view (**b**) and (**c**) lateral views.

We also calculated the deviation magnitude for specific facial landmarks directly on the 3D inspected meshes (Figure 7).

### 2.4. Statistical Analysis

Normal distribution of the data was established with the Shapiro–Wilk and Kolmogorov-Smirnov tests. To determine the method’s reliability, T_0_ and T_1_ images of 10 randomly selected patients were re-aligned and the measurements were recalculated one month later by the same investigator. Intra-examiner reliability was assessed to evaluate the reliability of the measurements in the same image and by the same investigator using the Intraclass Correlation Coefficient (ICC).

The random errors were calculated according to the Dahlberg’ formula (D =√ ∑ d^2^/2N) [27], where D is the error variance and d is the difference between the first and second measure. N is the sample size, which was re-measured. The systematic errors were also evaluated by comparing the first and second measurements using the dependent t test.

The (T_0_) and (T_1_) linear and angular measurements were compared. Significant differences at the level of 5% of significance were tested using a Wilcoxon signed rank test. In addition, for each patient, 3D deviation analysis was performed to calculate not only the maximum positive and negative deviation, but also the mean deviation for the facial meshes. Then, the Pearson correlation coefficient was calculated to determine the correlation between the expansion amount (expressed as the amount of jackscrew activation and resulting width difference of the upper arch) and the facial soft tissue changes. All statistical analyses were performed in SPSS 24.0 (IBM, Armonk, NY, USA).

## 3. Results

All parameters were normally distributed, according to the Shapiro–Wilk and Kolmogorov-Smirnov tests. No significant errors were found when repeating the measurements. The (ICC) values between the two sets of measurements were a high range of 0.821–0.979. The amount of random error was small enough (less than 0.5 mm/°), and no systematic errors were found between the measurements obtained on the two different occasions (*p* ≥ 0.05).

The mean value of the jackscrew activation after RME was 7.75 mm and the mean of the upper arch width difference calculated on the dental casts was 5.46 mm. Statistically significant changes of the soft tissue variables were found after RME using the Wilcoxon rank test (Table 3).

The mean linear changes of the nasal width and the nasal base width after RME were 1.02 mm and 1.21 mm, respectively, and 2.62 mm for the mouth width. While we also found significant angular changes after RME, the nasal tip angle increased by 3.2°, while the upper and lower lip angle have increased by 3.47° and 3.78°, respectively.

We also calculated the descriptive statistics of the maximum positive and negative deviation limits of the meshes, as shown in Table 4.

The mean of the maximum positive and negative deviation found in the total face were 3.09, −2.93 mm, respectively, 2.16, 2.81 mm both for the upper and lower face as a positive deviation, and −1.9, −2.87 mm for the negative ones. We also found 2.16, 1.37 mm as a positive deviation of both the upper and lower lip regions, and −1.5, −2.02 mm as the negative ones while the mean of the maximum positive and negative deviation found in the nasal region were 2.04, −1.25 mm, respectively.

Similarly, the mean, minimum, and maximum limits of the mean values were calculated in Table 5. While soft tissue changes were observed in the nasal and upper lip regions of 0.55, 0.53 mm, respectively, the changes observed in the total face region as well as the upper and lower lip regions were very small and almost neglected.

Changes were also observed at the level of the facial landmarks as seen in Table 6. The deviation found at the level of the right and left Alar points were 0.72 ± 0.45, 0.46 ± 0.59 mm, respectively. We also found changes at the level of Pronasal and Subnasal points of 0.44 ± 0.66, 0.66 ± 0.64 mm, and for the right and left Cheilion landmarks of 0.46 ± 1.621, 0.66 ± 1.98 mm, respectively.

**Figure 8:**
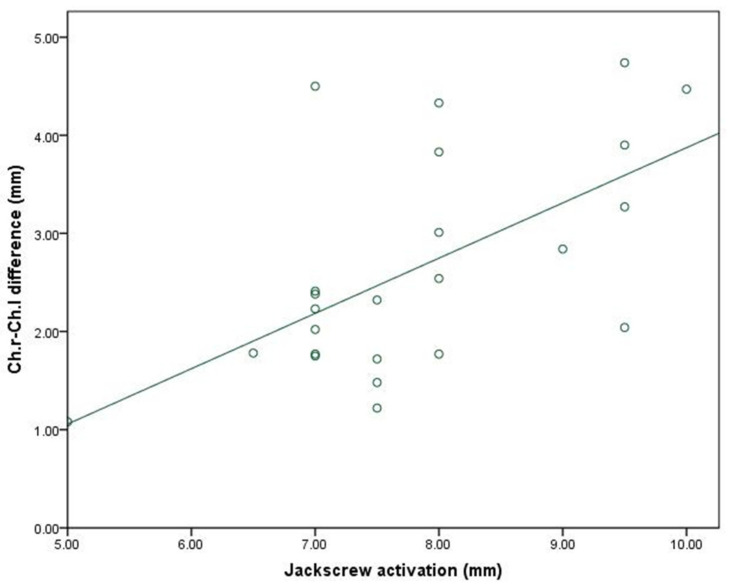
Scatter plot for the correlation between the mouth width difference and the jackscrew activation amount.

Moderate positive and negative correlations were found between the expansion amount and our variables using the Pearson correlation coefficient, but they did not reach the level of significance, except for the mouth width difference, which showed significant moderate positive correlation with the jackscrew activation at the level of 5% of significance, as seen in Figure 8.

## 4. Discussion

Assessment of the soft tissue changes after RME is a critical step during our orthodontic treatment. Various strategies, including laser scanners, stereophotogrammetry, and structured light scanners have recently been introduced for 3D soft tissue evaluation. It was found that the accuracy of the structured light scanner was at 0.57 ± 0.07 mm, and seemed to be the best in the midface region [28]. Jung et al. concluded that the accuracy of the structured light system is comparable to the direct anthropometric measurements. Furthermore, it was reported that more reliable 3D datasets are expected if the face of the subject was scanned from a couple of angles at the same time in less than 1 s [17]. Lee et al. investigated the reliability of 34 facial landmarks using a 3D handheld structured-light scanner (Artec Eva) and they observed constellations of landmarks that showed high reliability in each condition in terms of head posture and image resolution. The Artec EVA scanner is claimed to be comparable to other scanners mentioned in literature and leads to more accurate 3D models as compared to scanning with FaceScan3D [29].

Given the small sample size, the effects of patients’ age and sex could not be considered in this study. It was found in previous studies that the stage of maturation (based on the maturation of the cervical vertebrae) and sex had no significant effect on the soft tissue changes brought about by RME [30,31].

Our observations were made in a population of subjects who were in the active growth phase. It was presumed that growth might not cause a considerable interference with the studied parameters if evaluated for a period of 6 to 7 months [32]. Torun et al. found no significant difference between pre-pubertal and post-pubertal subjects [31]. This is in agreement with the outcomes of Johnson et al., who found that the developmental status had no significant effect on the soft tissue changes after RME [30]. Longo et al. proposed that the impact of growth is not a factor if a six-month observation period is used [33]. In their meta-analysis, Huang and co-workers came to the same conclusion [32]. Thus, in this study, a six-month retention period was applied.

To evaluate soft tissue changes, linear and angular measurements were made between point pairs and triads, completed with regional deviation analyses for various morphological regions of the face and for the facial landmarks.

### 4.1. Facial Soft Tissue Changes

Intercantal width increased by a mean of 0.65 mm after RME expansion. However, this increase did not reach a statistically significant level. Generally, forces generated with the aid of RME are claimed to affect circummaxillary sutures, along with the fronto-maxillary, nasomaxillary, and frontonasal ones [34,35,36]. The previously mentioned findings could provide an explanation for the 3D deviations observed around the eye. Baysal et al. suggested that the significant increase that they found in the intercanthal distance was the result of normal growth and development [37]. Dindaroglu and co-workers noted that, even if 3D facial images are captured rapidly, apparent 3D changes might occur in soft tissues around the eye because of the movement of the eyelids [26].

One of the most frequently examined anatomical regions in connection with RME is the nose, given the close anatomical relationship between the maxilla and the nasal area [26].

In this study, statistically significant increases were observed in nasal width (mean: 1.02 mm, *p* < 0.05), nasal base width (mean: 1.21 mm, *p* < 0.05), and nasal angle (mean: 3.2°, *p* < 0.05).

Berger and colleagues found a mean increase of 1.6 mm in nasal width, and a mean increase of 1 mm in nasal length after RME [10]. Furthermore, Altorkat and colleagues found that RME produces pyramidal expansion with the greatest transverse expansion at the anterior nasal spine landmark (ANS) [19]. However, the authors suggested that these changes may be neither symmetrical nor homogeneous when the anatomical relationship between the nose and maxilla is considered.

Pangrazio-Kulbersch and co-workers found a mean increase of 1.34 mm in alar width after RME [38], which is in accordance with our findings. Johnson et al. used direct measurements with an average of 7 mm of appliance expansion and found less than 1.5 mm change in nasal base and alar cartilage width, neither of which was clinically significant [30].

Increases were also observed in all transverse linear measurements in the nasal area including nasal base width, alar cartridge width, nasal tip retraction, and flattening of the nasal tip following RME. However, it was reported that these changes were very small and highly variable [19]. On the other hand, Silva Filho et al. used 2D photographs and concluded that RME did not cause changes in nasal morphology [39]. These observations markedly differ from our findings, and the reason for this difference most likely lies in the different scanning methods.

In this study, we found a statically significant increase (mean: 2.62 mm, *p* < 0.05) in mouth width after RME, likely because of the transverse expansion of the maxillary halves. We also found statically significant increases in the upper and lower lip angle (mean: 3.45° (*p* < 0.05) and 3.78° (*p* < 0.05), respectively).

Our results correspond to those of Altındi S et al. who also found a statistically significant increase in mouth width [18]. Similar changes in mouth width after RME were demonstrated in a recent CBCT study by Kim et al. [20]. On the contrary, the study of Baysal et al. found no statistically significant change in this respect [37], but this lack of significance might well be put down to the small sample size (17 subjects).

Despite the significant changes in both the linear and angular parameters found in the study of Dindaroglu F et al., it was considered that changes measured only in these dimensions may not reflect the actual soft tissue changes properly [26]. Thus, to complete linear and angular measurements, volumetric analyses were also conducted in the present study. A region was characterized by the mean value of all points of measurement within the given region.

We found significant facial soft tissue changes in both the nose and the upper lip regions (0.55 ± 0.26, 0.53 ± 0.67 mm, respectively), and we also noticed highly positive and negative deviations in the other facial morphological regions of the face, but the mean deviation for these regions changed only to a negligible extent.

The results of Dindaroglu et al. [26] are quite similar in this respect. The authors found that both positive and negative deviations were below 2 mm regardless of which morphological region of the face was examined. Specifically, the mean maximum positive and negative deviations for the nose area were 0.77 ± 0.34 and −0.94 ± 0.41 mm, respectively, and when all the points forming the nose region were considered, the mean deviation was 0.41 ± 0.21 mm, which is similar to our results.

Regarding the upper lip region, the mean positive and negative change in the same study was 0.87 ± 0.38 and −0.57 ± 0.14 mm, respectively, while the greatest recorded mean deviation was 1.44 mm [26].

The mean of the maximum positive deviation for the total face was 3.09 ± 0.92 mm in our study. It was 2.16 ± 0.77, 2.81± 0.87 mm for the upper and lower face regions, respectively, while the mean of the maximum negative deviation was −2.93 ± 0.85 mm for the total face, and −1.9 ± 0.79, −2.78 ± 0.89 mm for the upper and lower face regions, respectively.

Ong and co-workers, in a 3D study, reported that the mean maximum maxillary deviation was 1.2 ± 0.4 mm and 1.0 ± 0.3 mm for the right and left sides, respectively [23]. These results are quite close to our findings.

Although, in this study, the greatest mean deviations were recorded in the lower lip region (2.19 and 2.54 mm, positive and negative, respectively). The mean deviation for the whole sample in the lower lip region was negligible (−0.04 ± 1.24 mm).

For a more detailed understanding of the soft tissue alterations, deviations were calculated for specific facial landmarks. The greatest deviation was found at the alar point (0.72 ± 0.45 mm). Furthermore, the left alar point shifted by 0.46 ± 0.59 mm, and the subnasal point shifted by 0.66 ± 0.64 mm.

Kim et al. evaluated the deviations at different points in various nasal regions and detected a mean deviation of less than 1 mm for all the points, except for the ones in the sub-nasal region, where a mean deviation of 2.21 ± 1.23 mm was found [20]. The authors also found that the position of the left lip commissure changed by 0.65 mm, whereas the position of the right lip commissure changed by a mean of 1.20 mm [20]. Furthermore, we found that the position of the left and the right lip commissures changed by 0.66 and 0.46 mm, respectively, and also found a positive deviation at the pro-nasal point (0.44 mm) and negative deviation at the sublabial point (−0.43 mm). Kim et al. [20] found a positive change of 0.43 ± 1.24 mm at the nasion point, which did not change in our study.

Although we found positive and negative correlations between the amount of expansion and most of the facial soft tissue variables, these correlations did not reach a level of statistical significance. Only mouth width showed a moderately significant positive correlation with the amount of jackscrew activation (*p* < 0.05).

### 4.2. Limitations

A major limitation of this study is the small sample size, which prevented us from considering the effect of sex, among other factors, and it is also a limitation that prevents strong conclusions. Another relative limitation is the absence of a control group. However, based on the literature, we had reason to assume that, with a short observation period, it was not likely that normal growth would interfere with the results.

## 5. Conclusions

Significant changes in the nasal region and in the upper lip region were found after RME and six months of retention, and a significant positive correlation between mouth width and the amount of expansion was also observed.

The outcomes show that rapid expansion causes significant soft tissue changes on the surface of the face. While our results are a good starting point, further investigations with larger sample sizes and suitable controls are definitely necessary to allow generalizable statements about soft tissue responses after RME, especially in the long run.

## Figures and Tables

**Table 1:** Definition of the facial landmarks used in our study.

Landmark		Definition
Exocanthion	Ex *	Point at the outer commissure of the eye fissure
Endocanthion	End *	Point at the inner commissure of the eye fissure
Sellion	Se	The most posterior point of the frontonasal soft tissue contour in the midline of the base of the nasal root
Alare	Al *	The most lateral point on each alar contour (on the base view)
Pronasale	Prn	The most anterior midpoint of the nasal tip (on the right and left profile view). If a bifid nose is present, the more protruding tip is chosen to determine Pronasale
alar curvature point	Ac *	The point located at the facial insertion of each alar base. (on the submental view)
Subnasale	Sn	Midpoint on the nasolabial soft tissue contour between the Columella crest and the upper lip
Labiale superius	Ls	The midpoint of the vermilion line of the upper lip (on the submental view)
Stomion	Stm	The midpoint of the horizontal labial fissure
Chelion	Ch *	The point located at each labial commissure (on the frontal view)
Labiale inferius	Li	The midpoint of the vermilion line of the lower lip (on the right profile view)
Sublabiale	Sl	The most posterior midpoint on the Labiomental soft tissue contour that defines the border between the lower lip and the chin
Soft tissue Menton	Meˊ	The most inferior midpoint on the soft tissue contour of the chin located at the level of the 3-D cephalometric hard tissue Menton landmark

* Indicates bilateral landmarks (right and left).

**Table 2:** Definition of morphological regions used for the 3D deviation analyses.

**Region**	**Definition**
Total face	The facial region designated while creating masks prior to alignment
Upper face	The region between the line passing through the right and left Exocanthion points and the line passing through the Subnasal point parallel to that line
Lower face	The region between the line passing through the Subnasal point and the line passing through the Menton point parallel to that line
Upper lip	The region between the lines passing through the right and left Endocanthion points and the right and left Cheilion points, and the line passing through the Subnasal point
Lower lip	The region between the lines passing through the right and left Endocanthion points and the right and left Cheilion points, and the line passing through a Sublabiale point parallel to other lines
Nose	The region between the lines passing through the right and left Endocanthion points that are tangent to the nasal wings and the line passing through the Subnasal point

**Table 3:** Descriptive statistics of the pretreatment (T_0_) and post-treatment (T_1_) measurements.

		T_0_		T_1_		Δ = T_1_ − T_0_	*p*
		Mean	SD	Mean	SD		
Linear measurements (mm)							
Intercanthal width	(En_R_-En_L_)	30.93	2.42	31.58	2.4	0.65	NS
Nasal width	(Alar _R_.-Alar._L_)	31.98	2.83	33.09	3.27	1.02	0.023 *
Nasal base width	(Ac_R_-Ac_L_)	30.26	2.67	31.48	2.85	1.21	0.018 *
Mouth width	(ch_R_-ch_L_)	43.28	3.8	45.9	3.7	2.62	0.01 *
**Angular measurements (°)**							
Nasal tip angle	(Alar_R_-Prn-Alar_L_)	85.82	6.13	89.03	6.2	3.2	0.05 *
Upper lip angle	(Ch_R_-ls-Ch_L_)	110.66	4.3	114.13	5.7	3.47	0.023 *
Lower lip angle	(Ch_R_-li-Ch_L_)	122.16	6.1	125.94	6.34	3.78	0.047 *

* Significant changes at the level of 5% level of significance (α < 0.05) based on the Wilcoxon signed rank test. NS, Not significant.

**Table 4:** Descriptive statistics of the maximum positive and negative deviation in the morphological regions.

Morphological Region	Maximum Positive Deviation Limits (mm)				Maximum Negative Deviation Limits (mm)			
	Minimum	Maximum	Mean	SD	Minimum	Maximum	Mean	SD
Total face	1.72	5.43	3.09	0.92	−5.88	−1.93	−2.93	0.85
Upper face	0.97	3.9	2.16	0.77	−3.5	−0.89	−1.9	0.79
Lower face	1.47	5.22	2.81	0.87	−5.88	−1.34	−2.78	0.89
Nose	0.68	3.9	2.04	0.71	−3.49	−0.47	−1.25	0.67
Upper lip	0.64	5.06	2.16	0.97	−5.69	0.11	−1.5	1.03
Lower lip	−0.63	5.22	1.37	1.16	−4.68	0.53	−2.02	1.67

**Table 5:** Descriptive statistics of the mean deviation in the morphological region.

Morphological Region	Minimum	Maximum	Mean	SD
Total face	−0.25	0.12	−0.02	0.08
Upper face	−0.99	0.65	0.06	0.3
Lower face	−0.54	0.32	−0.02	0.16
Nose	−0.1	0.96	0.55	0.26
Upper lip	−0.87	1.79	0.53	0.67
Lower lip	−2.54	2.19	−0.04	1.24

**Table 6:** Deviation analysis of the facial soft tissue landmarks.

Landmark		Deviation Mean (mm)	SD
Endocanthion (right)	End_r_	0.28	0.39
Endocanthion (left)	End_l_	0.25	0.55
Sellion	Se	−0.03	0.41
Alar point (right)	Alar_r_	0.72	0.45
Alar point (left)	Alar_l_	0.46	0.59
Pronasal	prn	0.44	0.66
Subnasal	Sn	0.66	0.64
Cheilion (right)	Ch_r_	0.46	1.62
Cheilion (left)	Ch_l_	0.66	1.98
Labiale superius	Ls	0.23	1.36
Labiale inferius	Li	0.2	1.37
Stomion	Sto	−0.11	1.71
Sublabiale	Sl	−0.43	1.31
Soft tissue menton	meˊ	0.02	0.89

## Data Availability

Data used in this study are available from the corresponding author upon a reasonable request.
